# Level of Acceptance of Illness and Its Association with Quality of Life among Patients with Epilepsy in North Shewa, Ethiopia

**DOI:** 10.1155/2022/1142215

**Published:** 2022-09-12

**Authors:** Yonas Teshome, Yerukneh Solomon, Feredegn Talargia, Negese Worku, Abreham Shitaw, Abebaye Aragaw Leminie

**Affiliations:** ^1^Department of Biomedical Sciences, College of Medicine, Debre Berhan University, Debre Berhan, Ethiopia; ^2^Department of Internal Medicine, College of Medicine, Debre Berhan University, Debre Berhan, Ethiopia; ^3^Department of Physiology, College of Health Sciences, Addis Ababa University, Addis Ababa, Ethiopia

## Abstract

Acceptance of illness is regarded as an indicator of functioning and predictor of quality of life. However, quality of life of patients with epilepsy in sub-Saharan countries worsen because of low medication adherence, increased morbidity and mortality, and the stigmatization associated with the disease. This research is aimed at assessing the level of acceptance of illness of patients with epilepsy and associated quality of life in North-East Ethiopia. *Methods*. A cross-sectional study was conducted from January to June 2021 at the Debre Berhan Referral Hospital, North-East Ethiopia. A total of 78 patients with epilepsy aged more than 18 years were randomly selected and assessed using Quality of Life in Epilepsy Inventory 31 and acceptance of illness scale. In addition, authors owned questionnaire were used to evaluate the sociodemographic and clinical characteristics of the patients. *P* value < 0.05 at 95% confidence level was considered to be statistically significant in all the analysis. *Result*. The study participants' age varied between 18 and 67 years with the mean age of 28.9 years. Phenobarbital was the most used (73.9%) antiepileptic drug, and 68.7% (*n* = 66) of the patients seizure was controlled. 72.9% (*n* = 70) of the patients had medium acceptance of illness (scored 20-30), while 17.7% (*n* = 17) had low illness acceptance level (scored 8-19), and 9.4% (*n* = 9) had high acceptance of illness (scored 31-40). The mean of overall acceptance of illness among epileptic patients was 21.04 ± 7.21. The overall score of QOLIE-31 was 79.14 ± 25.46, and the highest mean score was for cognitive (83.5 ± 27.1), while the lowest mean score was that of medication effect (72.7 ± 28.7). Five of the seven QOLIE-31 components correlated significantly with level of acceptance of illness. Cognitive domain (*r* = 0.498, *p* < 0.001) demonstrated the highest correlation followed by overall quality of life (*r* = 0.489, *p* < 0.001), seizure worry (*r* = 0.433, *p* < 0.001), energy/fatigue (*r* = 0.342, *p* < 0.001), and emotional well-being (*r* = 0.278, *p* < 0.001). *Conclusion*. Patients with epilepsy in the study area had medium acceptance of illness, and nearly half of them had mean and more than the mean quality of life. The patients' acceptance of illness was significantly associated with overall quality of life, seizure worry, emotional well-being, and cognitive domain of the patients.

## 1. Background

Epilepsy is a chronic noncommunicable disease of the brain which affects around 50 million people world widely [1], and about 80% of epileptic patients are living in resource poor countries [2]. A further 500 million people (including families and care givers) are thought to be indirectly affected by the disease [3]. In developed nations, epilepsy occurs with an annual incidence ranging from 20 to 70 cases per 100,000, while the incidence of epilepsy in developing countries may be as high as 190 per 100, 000 people [4]. However, its burden in sub-Saharan African countries worsen because of low medication adherence and belief [8], increased morbidity and mortality [5], and the stigmatization associated with the disease [6, 7].

The incidence and prevalence of epilepsy in Ethiopia were reported to be 64/100,000/year and 5.2/1000, respectively [8], but it can reach a prevalence of 29.5/1000 in Zay society of Ethiopia [9]. The morbidity and mortality associated with the disease in low-income countries increase because of the shortage of trained health workers, insufficient antiepileptic drug supplies, and limited diagnostic equipment. Furthermore, many patients with epilepsy in low-income countries do not seek medical treatment due to cultural issues and economic reasons [10, 11].

Epilepsy imposes about 0.5% of the global burden of disease causing substantial socioeconomic problem on the patients [1]. However, the personal burden of illness cannot be described fully by measures of diseases only; it needs measurement of health-related quality of life which goes beyond direct manifestations of illness to study patients' personal morbidity [12]. And studies shows a significant correlation between acceptance of illness and quality of life of patients [13–15].

Acceptance of chronic illness refers to psychological adjustment for the chronic illness [16].The level of chronic illness acceptance is an indicator of functioning and predictor of quality of life; and the greater the acceptance of disease, the less mental discomfort and less severe negative emotions [17]. But in patients with epilepsy, stigma associated with illness and psychosocial factors is causing poor physical as well as psychological well-being [18] with few interventions being implemented to change illness perceptions [19]. Therefore, this study is aimed at assessing the level of acceptance of illness of patients with epilepsy and their associated quality of life in North-East Ethiopia.

## 2. Methods

### 2.1. Study Period and Area

The study was conducted at the psychiatry unit of the Debre Berhan Referral Hospital, North-East Ethiopia, from September to June 2021.

### 2.2. Study Design

Hospital-based cross-sectional study was conducted.

### 2.3. Source Population

All patients diagnosed with epilepsy at the Debre Berhan Referral Hospital are the source population.

### 2.4. Study Population

All epileptic patients aged more than 18 years and who had been living with epilepsy for at least one year are the study population.

### 2.5. Eligibility Criteria

Age greater or equal to 18 years old epileptic patients who lived with epilepsy for at least one year and gave informed consent were included in the study. Patients less than 18 years old, patients diagnosed with epilepsy earlier (before a year), and those who did not gave informed consent were excluded from the study.

### 2.6. Sample Size Determination

There is no research done about the magnitude of epilepsy in North Shewa zone. Therefore, *p* = 3% was taken from the study done on Zay society, Ethiopia, to determine the representative sample size. Using single population proportion formula,

(1) $$\begin{array}{c} P=0.03\;Q=1-p=0.97\\ Z=2.58\\ n=\frac{{\left(Z_{\alpha /2}\right)}^{2}P\left(1-P\right)}{d^{2}}=\frac{{\left(2.58\right)}^{2}\left(0.03\times 0.97\right)}{{\left(0.05\right)}^{2}}=78\text{ adding nonresponse rate}=98. \end{array}$$

### 2.7. Data Collection Procedure

The data was collected by trained psychiatric nurse at the Debre Berhan Referral Hospital using authors owned questionnaire, standard acceptance of illness scale (AIS) by B.J. Felton et al., and Quality of Life in Epilepsy Inventory 31. The AIS enables researchers to assess patients' acceptance level of any disease, and its statements are presented in accordance to the Likert technique by which respondents' agreement or disagreement (set of attitude statements) can be expressed with the use of a five-point scale (1-strongly agree, 2-agree, 3-neutral, 4-disagree, and 5-strongly disagree). This five-point Likert scale is an interval scale, and when the mean response is from 1 to 1.80, it means strongly agree; from 1.81 to 2.60, it means agree; from 2.61 to 3.40, it means neutral; from 3.41 to 4.20, it means disagree; and from 4.21 to 5.00, strongly disagree.

The sum total score between 8 and 40 points is a measure of illness acceptance. The higher the score, the better the illness adaptation and the lower mental discomfort of the patient. Scores below 20 points are considered low and indicate no or poor acceptance and adaptation to the disease as well as significant emotional problem related to it. Scores between 20 and 30 points indicate moderate level of acceptance of the illness, and scores more than 30 points indicate high or full level of acceptance of the disease.

Additionally, quality of life of the patients was assessed using Quality Of Life In Epilepsy Inventory 31 (QOLIE-31) which contains seven multi-item scales that tap the following seven health concepts: emotional well-being (5 items), social functioning (5 items), energy/fatigue (4 items), cognitive functioning (6 items), seizure worry (5 items), medication effect (3 items), and overall quality of life (2 items). Clinical data including seizure control, seizure frequency, length of antiepileptic drug treatment, and antiepileptic drug used for treatment were assessed by face-to-face interview using authors' questionnaire and then cross-checked with patients' medical record.

### 2.8. Ethical Approval and Consent to Participate

The study was conducted in accordance with the guidelines laid down in the Declaration of Helsinki, and all the procedures were approved by the Ethical Committee of the College of Medicine, Debre Berhan University, and Debre Berhan Referral Hospital Ethical Committee. Written informed consent was obtained from all subjects before participation.

### 2.9. Statistical Analysis

Data were coded, checked, and entered into Epi-data statistical software version 3.1 and then exported to SPSS software version 23 for analysis. Descriptive statistics were presented as frequency and percentage. Pearson's correlation was used to compute the association between dependent and independent variables. *P* value < 0.05 at 95% confidence level was considered to be statistically significant in all the analysis.

## 3. Results

### 3.1. Sociodemographic and Clinical Characteristics

The study participants' age varied between 18 and 67 years with the mean age of 28.9 years. The majority of the study participants were male (63.5%), single (50%), and educated up to primary school (36.5%). Phenobarbital was the most used (73.9%) antiepileptic drug, and in about 68.7% (*n* = 66) of the patients, seizure was controlled (Table 1).

### 3.2. Acceptance of Illness Scale

Seventy-two point nine percent (*n* = 70) of the patients had medium acceptance of illness (scored 20-30), while 17.7% (*n* = 17) had low illness acceptance level (scored 8-19). But only 9.4% (*n* = 9) had high acceptance of illness (scored 31-40). The mean of overall acceptance of illness among epileptic patients was 21.04 with standard deviation of 7.21, which indicates medium level of acceptance of a disease among epileptic patients in the study area (Table 2).

### 3.3. Quality of Life of Epileptic Patients

The overall score of QOLIE-31 was calculated after multiplying subscale total scores by their respective weight. Accordingly, the overall score of QOLIE-31 among epileptic patients in the study area was 79.1 ± 25.4, and the highest mean score was for cognitive (83.5 ± 27.1), while the lowest mean score was that of medication effect (72.7 ± 28.7). Nearly half of the patients 47 (48.96%) had an overall quality of life score greater or equal to the mean score level, while the remaining 49 (51.04%) had overall quality of life score below the mean (Table 3).

Five of the seven QOLIE-31 components correlated significantly with level of acceptance of illness. Cognitive domain (*r* = 0.498, *p* < 0.001) demonstrated the highest correlation followed by overall quality of life (*r* = 0.489, *p* < 0.001), seizure worry (*r* = 0.433, *p* < 0.001), energy/fatigue (*r* = 0.342, *p* < 0.001), and emotional well-being (*r* = 0.278, *p* < 0.001). The correlation matrix was assessed for the multicollinearity between the quality of life subscale items, and no multicollinearity was observed, or no variables shows a correlation coefficient of >0.70. Additionally, for all variables, the tolerance value shows >0.1 and variance inflation factor (VIF < 10), which confirms absence of multicollinearity effect between variables (Table 4).

## 4. Discussion

The finding of this study shows that patients in the study area had medium level of acceptance of illness. The mean value of the overall acceptance of illness in this study is in line with the study done in Poland [20]. However, small percentage of the study participants in this study area had higher acceptance of illness compared to the aforementioned study. This might be due to increased morbidity, mortality, and the stigmatization associated with the disease in low economic country [8, 21].

Although there are no sufficient studies about acceptance of illness scale of epileptic patients for comparison, low acceptance of illness was reported by studies on chronic disease conditions like cancer [22], chronic respiratory diseases [23, 24], and peripheral diabetic neuropathy [25]. Medium acceptance of illness was reported with lung cancer [26], type II diabetes mellitus [27], colorectal cancer [28], and breast cancer [29]. There are also studies which reported higher acceptance of illness among renal transplantation patients [30] and pregnant women with hyperglycemia [31]. Some studies also indicated a strong relationship between illness acceptance and quality of life [32], and the extent the patients accepts their chronic diseases has shown to impact their quality of life [33].

Compared with the study done using QOLIE-31 tool, the mean quality of life of epileptic patients in this study area is similar with a study done in Mekelle [34]. Highest mean value was reported for medication effect in the study done in Mekelle, while medication effect had the lowest mean score in this study. The discrepancy might be due to variation in the selection of drugs for treatment, which in the case of this study was conventional antiepileptic drugs (phenobarbital for more than 70% of the patients) resulting in higher adverse effect and low quality of life score [35].

The mean total QOLIE 31 score in this study was lower than study done in Wollo [36] and higher than the study done at Jimma [37]. Additionally, about half of the patients had an overall quality of life score greater or equal to the mean score level, while the remaining half had overall quality of life score below the mean. This is also consistent with studies from Wollo, Jimma, Gondar [38], and Addis Ababa [39]. This might be due to socioeconomic and demographic similarity between the study areas.

It has been shown that the level of acceptance of illness had significant association with health-related quality of life in different studies [40]. Similarly, the finding of this study shows a significant correlation between level of acceptance of illness and quality of life in epileptic patients. Accordingly, cognitive domain demonstrated the highest correlation followed by overall quality of life, seizure worry, energy/fatigue, and emotional well-being. This justifies level of acceptance of illness to be an important element in the quality of life of patients and a self-rated health of patients [40, 41]. Therefore, to add an important element in the holistic medical or nonmedical care, educating epileptic patients about their chronic disease might be essential to increase patients' level of acceptance of illness and thereby increase their quality of life [42, 43].

## 5. Conclusion

Patients with epilepsy in the study area had medium acceptance of illness, and nearly half of them had mean and more than the mean quality of life. The patients' acceptance of illness was significantly associated with overall quality of life, seizure worry, energy/fatigue, emotional well-being, and cognitive domain of the patients.

## Figures and Tables

**Table 1:** The sociodemographic characteristics of patients with epilepsy at the Debre Berhan Referral Hospital, 2020/2021.

Sociodemographic variable (*N* = 96)	Frequency	Percent (%)
Age		
18-25	35	36.4
26-34	30	31.3
35-44	16	16.7
≥45	15	15.6
Sex		
Male	61	63.5
Female	35	36.5
Marital status		
Single	48	50
Married	44	45.8
Divorced	4	4.2
Educational status		
Unable to read and write	26	27.1
Able to read and write	10	10.4
Primary school	35	36.5
Secondary school	15	15.6
Diploma and above	10	10.4
Seizure control		
Controlled seizure	66	68.7
Uncontrolled seizure	30	31.3
Seizure frequency		
Daily	3	3.1
Weekly	9	9.4
Monthly	18	18.8
Less than once a month	66	68.7
Length of AED treatment		
1-5 years	51	53.1
6-10 years	25	26.1
11-15 years	7	7.3
16-20 years	5	5.2
≥21 years	8	8.3
AEDs used for treatment		
Phenobarbital	71	73.9
Carbamazepine	12	12.5
Sodium valproate	6	6.3
Phenytoin	5	5.2
Other	2	2.1

AEDs: antiepileptic drugs.

**(a):** 

Level of illness acceptance	Score	Frequency	Percent (%)
Low acceptance	8-19	17	17.7
Medium acceptance	20-30	70	72.9
High acceptance	>30	9	9.4

**(b):** 

Over all acceptance of illness			
Minimum	Maximum	Mean	Standard deviation
9.00	35.00	21.04	7.21

**Table 3:** QOLIE-31 score of patients with epilepsy at the Debre Berhan Referral Hospital, 2020/2021.

QOLIE-31 subscales	Final score	Weight	Subtotal
Seizure worry	80.65377	.8	6.45
Overall quality of life	77.5	.14	10.85
Emotional well-being	77.7904	.15	11.67
Energy/fatigue	78.438	.12	9.41
Cognitive	83.4769	.27	22.54
Medication effect	72.775	.03	2.18
Social functioning	76.3741	.21	16.04
QOLIE-31 score: 6.45 + 10.85 + 11.67 + 9.41 + 22.54 + 2.18 + 16.04 = 79.14 ± 25.46			

**Table 4:** Partial correlation (Pearson's *r*) among the means of QOLIE-31 subscales and acceptance of illness, 2020/2021.

Variables	1	2	3	4	5	6	7	8
1. Acceptance of illness								
2. Seizure worry	.433^∗∗^							
3. Overall quality of life	.489^∗∗^	.393^∗∗^						
4. Emotional well-being	.278^∗∗^	.147	-.096					
5. Energy/fatigue	.342^∗∗^	.247^∗∗^	.028	.665^∗∗^				
6. Cognitive domain	.498^∗∗^	.408^∗∗^	.380^∗∗^	.337^∗∗^	.246^∗∗^			
7. Medication effect	.112	.265^∗∗^	.119	-.114	-.204^∗^	-.094		
8. Social functioning	-.086	.051	.067	.176∗	.038	.120	.138	

Significant at ^∗^*p* < 0.05 and ^∗∗^*p* < 0.001; 1-8 in the column represent each variables listed in the rows, respectively.

## Data Availability

The dataset used and analyzed during this study are available from the corresponding author on reasonable request.

## Additional Points

*Limitation*. Lack of assessment of epilepsy-associated comorbidities and financial status of the study participants might greatly influence the quality of life.
